# Butein Inhibits Oxidative Stress Injury in Rats with Chronic Heart Failure via ERK/Nrf2 Signaling

**DOI:** 10.1155/2022/8684014

**Published:** 2022-01-04

**Authors:** Peng Liu, Quanli Pan

**Affiliations:** ^1^Department of Cardiovascular Medicine, Wuhan Hospital of Traditional Chinese Medicine, Wuhan, 430000 Hubei, China; ^2^Department of Emergency Treatment, Wuhan Hospital of Traditional Chinese Medicine, Wuhan, 430000 Hubei, China

## Abstract

**Background:**

Chronic heart failure (CHF) is a serious heart disease resulting from cardiac dysfunction. Oxidative stress is an important factor in aging and disease. Butein, however, has antioxidant properties. To determine the effect of butein on oxidative stress injury in rats, a CHF rat model was established.

**Methods:**

The CHF rat model was induced by abdominal aortic coarctation (AAC). Rats in CHF+butein and sham+butein group were given 100 mg/kg butein via gavage every day to detect the effect of butein on oxidative stress injury and myocardial dysfunction. The cardiac structural and functional parameters, including the left ventricular end-systolic dimension (LVESD), the left ventricular end-diastolic dimension (LVEDD), the left ventricular ejection fraction (LVEF), and the left ventricular fractional shortening (LVFS), were measured. Oxidative stress was measured through the production of reactive oxygen species (ROS), superoxide dismutase (SOD), glutathione peroxidase (GSH-Px), catalase (CAT), and malondialdehyde (MDA). Cardiac injury markers like creatine kinase-MB (CK-MB), lactate dehydrogenase (LDH), and aspartate aminotransferase (AST) were evaluated. Hematoxylin and eosin (H&E) staining was used to observe the myocardial cell morphology. The effect of butein on the extracellular signal-regulated kinase (ERK)/nuclear factor-E2 p45-related factor (Nrf2) signaling was confirmed by Western blot analysis.

**Results:**

Butein had a significant effect on CHF in animal models. In detail, butein inhibited oxidative stress, relieved cardiac injury, and alleviated myocardial dysfunction. Importantly, butein activated the ERK1/2 pathway, which contributed to Nrf2 activation and subsequent heme oxygenase-1 (HO-1) and glutathione cysteine ligase regulatory subunit (GCLC) induction.

**Conclusions:**

In this study, butein inhibits oxidative stress injury in CHF rat model via ERK/Nrf2 signaling pathway.

## 1. Introduction

As a common chronic cardiovascular disease, chronic heart failure (CHF) has a high mortality rate of incidence, which is up to 20% [1–3]. Because of aging of the population and the increase in the prevalence of CHF in the elderly, CHF has become a growing public health problem [4]. CHF is mainly caused by myocardial infarction, hypertension, valvular heart disease, and cardiomyopathy. Furthermore, oxidative stress has been proved to aggravated CHF [5–7], and the leading cause of oxidative stress was the imbalance between the production of reactive oxygen species (ROS) and the endogenous antioxidant defense system [8–12]. However, the prevention and existing treatment strategies of oxidative stress in CHF are not effective in improving the survival rate, so further studies are urgently needed.

Traditional Chinese medicine has been used for alleviating different diseases for a long time and is generally considered a multitarget therapy with minimal adverse actions [13–16]. Chalcones is a subclass of flavonoids with an open C-ring structure. Chalcone and its derivatives have been reported to have various biological activities [17]. As a chalcone derivative and a plant polyphenol from the heartwood of *Dalbergia odorifera*, *Caragana jubata*, and *Rhus verniciflua* Stokes, butein (2′,3,4,4′-tetrahydroxychalcone) has been identified to have wide range of pharmacological effects, such as antioxidant, anticancer, anti-inflammatory, and antimicrobial [18–21]. Studies have shown that Butein can effectively reduce lipid peroxidation and superoxide anion production in rat liver microsomes by its inherent antioxidant capacity, thus preventing free radical-induced cytotoxicity [22]. Butein also protects rat primary hepatocytes from oxidative damage by promoting glutamate cysteine ligase expression and glutathione levels [23]. However, it is unknown whether butein has such antioxidant capacity in CHF; thus, we decided to study the function and mechanism of butein in CHF rats.

Nuclear factor- (erythroid-derived 2-) like 2 (Nrf2) acts as a redox sensitive transcription factor to conduct the antioxidant defense system [24]. Nrf2 in nucleus then promotes transcription of heme oxygenase-1 (HO-1), which is the antioxidant response element, and protects the cell defense against oxidative stress [25–29]. Previous studies have shown that extracellular signal-regulated kinase (ERK) 1/2 is an important participant in oxidative and endoplasmic reticulum stress [30]. Importantly, ERK1/2 is also a significant activator of Nrf2 [31]. Based on the evidence, a hypothesis was proposed that butein may affect oxidative stress injury of myocardial cells via ERK/Nrf signaling pathway in CHF rat model.

In this study, experiments were designed to examine the effects of butein on oxidative stress injury of CHF rats. We have also studied the downstream pathway of butein in CHF rats for the first time, which may bring new targets or reference significance for improving outcomes of CHF.

## 2. Material and Methods

### 2.1. Animals

Sixty male Sprague–Dawley rats (8–12 weeks of age) with 220–260 g weight range were prepared from Beijing HFK Biotechnology Co., Ltd. (SCXK-2018-0004) and raised in a controlled environment (21~25°C, 40~45% humidity, and 12 h light/dark cycles). They were provided with free drinking water and standard chow diet according to the guidelines for the Care and Use of Laboratory Animals. Animal Care and Use Committee of Wuhan Hospital of Traditional Chinese Medicine (Hubei, China) approved all animal experimental procedures. All animals received humane care in compliance with Institutional Animal Care and Use Committees of Wuhan Hospital of Traditional Chinese Medicine. Use of animals in this study was confirmed with the Guide for the Care and Use of Laboratory Animals published by the US National Institutes of Health (the 8th Edition).

### 2.2. Experimental Design and Model Establishment

Abdominal aortic coarctation (AAC) establishes a pressure overload CHF model. Its mechanism is that by narrowing the abdominal aorta, aortic pressure increases, cardiac afterload increases, myocardial compensatory hypertrophy, ventricular volume increases, heart expands, cardiac decompensation in the later stage, resulting in myocardial function and structural damage, and finally, heart failure. AAC model in rats was established by referring to the reference method [32]. The steps were as follows. After intraperitoneal injection of 2% sodium pentobarbital at 0.2 mL/100 g (Sigma-Aldrich, St. Louis, MO, United States) and preparation of the skin (shaved), the rats were fixed on the operating table supine. AAC was established using a 7-0 suture tied around the abdominal aorta in which a 20-gauge needle was inserted. The needle was then retracted yielding a 70% constriction with an outer aortic diameter of approximately 0.9 mm. In the sham operation group, the same surgery was performed as described above except that the aorta was not constricted. All animals had free access to water and a standard rodent chow. The rats were then divided into four groups (*n* = 15/group): sham+ phosphate buffer saline (PBS), CHF+PBS CHF+butein and sham+butein groups. Rats in sham+PBS and CHF+PBS group were orally administrated with water via gavage and were intraperitoneally administrated with PBS. Rats in CHF+butein and sham+butein group were given 100 mg/kg butein via gavage every day. The rats were treated for a total of 6 weeks. At the end of experiment, the rats were euthanized by an intraperitoneal injection of sodium pentobarbital (100 mg/kg), and blood samples and myocardial tissue were collected, and the hearts were immediately isolated. Blood samples were stored at −20°C; the heart and myocardial tissue were stored at −80°C for analysis.

### 2.3. Examination of Cardiac Structure and Function

The left ventricular end-systolic dimension (LVESD), left ventricular end-diastolic dimension (LVEDD), left ventricular fractional shortening (LVFS), and left ventricular ejection fraction (LVEF) were performed using echocardiography imaging using a Vevo 2100 high-resolution imaging system (MS250; Visual Sonics, Toronto, Canada).

### 2.4. Measurement of Antioxidative Enzymes

Evaluation of glutathione peroxidase (GSH-Px), superoxide dismutase (SOD), malondialdehyde (MDA), and catalase (CAT) in myocardial homogenates were carried out using commercial kits (Nanjing Jiancheng Bioengineering Institute, Nanjing, China). The mixture was placed in a water bath at 37°C for 30 min to detect SOD activity. Then, the absorbance value was measured at 560 nm. The GSH-px activities were detected at a wavelength of 412 nm according to the produced enzyme-catalyzed reaction product. CAT activity was measured in absorbance at 240 nm. The MDA content was measured at a wavelength of 532 nm.

### 2.5. Evaluation of Cardiac Injury Markers

Blood samples (0.5 mL) were collected from the rats at the end of the study; the sera were separated and stored at −20°C for measuring cardiac damage markers aspartate aminotransferase (AST), creatine kinase-MB (CK-MB), and lactate dehydrogenase (LDH) with commercial kits from Nanjing Jiancheng Bioengineering (Nanjing, China).

### 2.6. Heart Mass Index and Left Ventricular Mass Index

The mass of the whole heart (WHM) and the left ventricle (LVM) was measured. Then, the ratios of WHM or LVM to body mass (WHM/BM, LVM/BM) were calculated as the heart mass index (HMI) and left ventricular mass index (LVMI) after the rats were sacrificed.

### 2.7. Histological Examinations

After fixation for 72 h, the myocardial tissue was dehydrated and embedded in paraffin, and samples were sliced into pathological sections (4 *μ*m) according to standard procedures [33]. Hematoxylin-eosin (H&E) was used to stain pathological sections from the same rat. A light microscope (Olympus Medical Systems Corp, Tokyo, Japan) was employed to observe H&E staining sections. Then, the myocyte cross-sectional area of different groups was measured to confirm whether there was a difference amongst four experimental groups in the myocardial morphology.

### 2.8. Western Blot Analysis

A total of 30 *μ*g protein was separated by sodium dodecyl sulfate polyacrylamide gel electrophoresis (SDS-PAGE) and electrotransferred onto polyvinylidene fluoride (PVDF) membrane (Millipore, USA). The membrane was incubated overnight with primary antibody after blocking with 5% skim milk. The primary antibodies were provided by Abcam Company, including anti-ERK (ab32537, 1 : 1000), anti-phospho-ERK (ab201015, 1 : 1000), anti-Nrf2 (ab62352, 1 : 1000), anti-polyadenosine-diphosphate-ribose polymerase (PARP) (ab120981, 1 : 1000), anti-GAPDH (ab8245, 1 : 1000), anti-HO-1 (ab52947, 1 : 1000), and anticatalytic subunit of glutamylcysteine ligase (GCLC) (ab207777, 1 : 1000). Membranes were subsequently incubated with appropriate HRP-conjugated secondary antibody at room temperature for 1 h. The bands were visualized using an enhanced chemiluminescence kit (PerkinElmer Life Science, Boston, MA, USA) and were scanned by a luminescence image analyzer (Fuji Film LAS-4000, Japan). The bands were measured with Image Gauge software.

### 2.9. Statistical Analyses

SPSS 22.0 (Armonk, New York, USA) was used for statistical analyses. The results are expressed as the mean ± standard deviation for each group. For multiple comparisons, analysis of variance (ANOVA) was used. And for two groups, Student's *t*-test was conducted. *p* < 0.05 was considered significant.

## 3. Results

### 3.1. Effects of Butein on Animal Models of CHF

**Figure 1:**
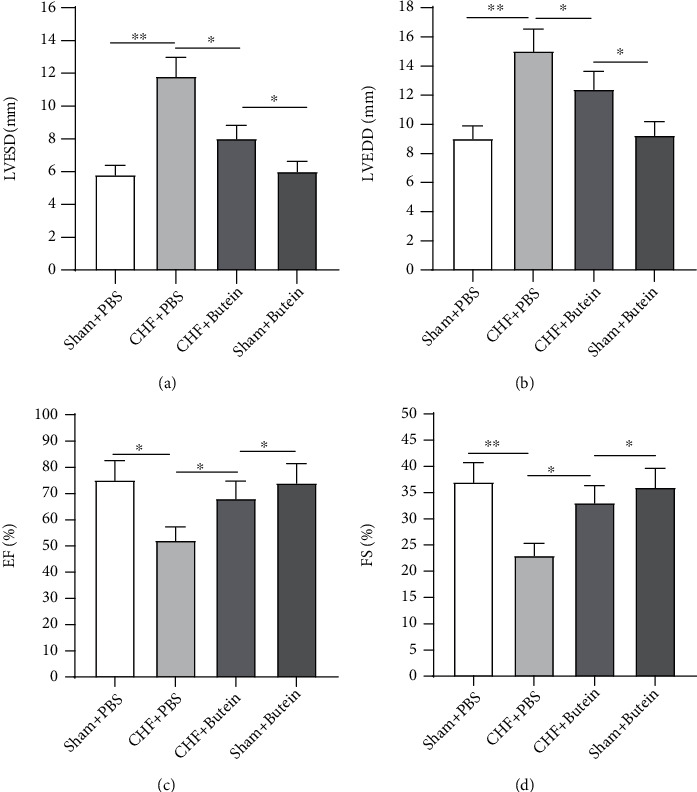
Effects of butein on animal models of CHF. (a–d) The values of echocardiographic parameters were measured and compared between experimental groups. ^∗^*p* < 0.05, ^∗∗^*p* < 0.01.

After the establishment of CHF model, the cardiac function was assessed. LVESD and LVEDD doubled in CHF+PBS group, compared with those in sham+PBS group, while butein partially reversed the increase in CHF+PBS group (Figures 1(a) and 1(b)). Inversely, LVEF and LVFS were reduced in CHF+PBS group, compared with those in sham+PBS group, while butein restored LVEF and LVFS to normal levels, suggesting the protective role of butein in CHF (Figures 1(c) and 1(d)). These results indicated that CHF models are successfully established, and butein exerts a protective effect on animal models of CHF.

### 3.2. Butein Alleviates Oxidative Stress in the Heart

**Figure 2:**
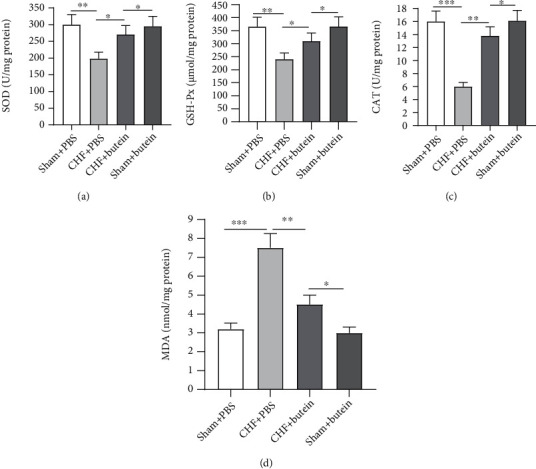
Butein alleviates oxidative stress in the heart. (a–d) The superoxide dismutase (SOD), glutathione peroxidase (GSH-Px), catalase (CAT), and malondialdehyde (MDA) levels were assayed by kits. ^∗^*p* < 0.05, ^∗∗^*p* < 0.01, ^∗∗∗^*p* < 0.001.

To reveal the effect of butein on oxidative stress, the levels of SOD, GSH-Px, CAT, and MDA were measured in heart tissues. The levels of SOD, GSH-Px, and CAT showed a reduction in CHF+PBS group compared with those in sham+PBS group. However, the levels of SOD, GSH-Px, and CAT were promoted in CHF+butein, compared with those in CHF+PBS group (Figures 2(a)–2(c)). In addition, the content of MDA in CHF+PBS was higher than that in sham+PBS, while butein partially reversed the upregulation in CHF+PBS and decreased the content of MDA (Figure 2(d)). These results implied that butein ameliorated oxidative stress of CHF rats.

### 3.3. Butein Relieves Cardiac Injury

**Figure 3:**
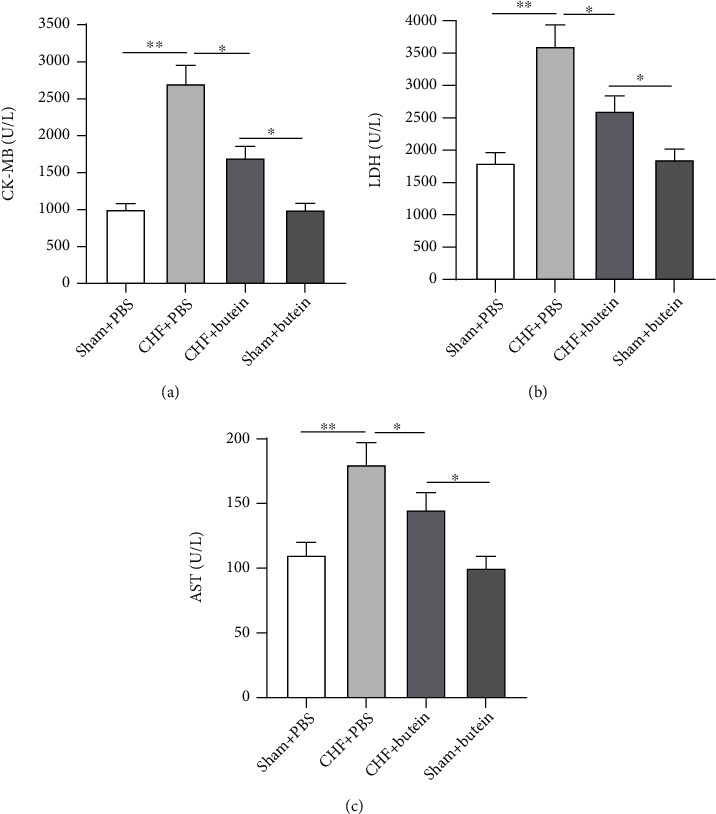
Butein relieves cardiac injury. (a–c) The cardiac damage markers like creatine aspartate aminotransferase (AST), kinase-MB (CK-MB), and lactate dehydrogenase (LDH) were measured using commercial kits. ^∗^*p* < 0.05, ^∗∗^*p* < 0.01.

To assess the extent of myocardial damage, changes in serum myocardial enzymes (CK-MB, LDH, and AST) were measured at 6 weeks of treatment. The levels of CK-MB, LDH, and AST were almost two times higher in CHF group than those in sham operation group. However, compared with the CHF+PBS group, butein partially restored the upregulation in these serum myocardial enzyme levels (Figures 3(a)–3(c)). These results suggested that butein relieved cardiac injury.

### 3.4. Butein Relieves Myocardial Dysfunction

**Figure 4:**
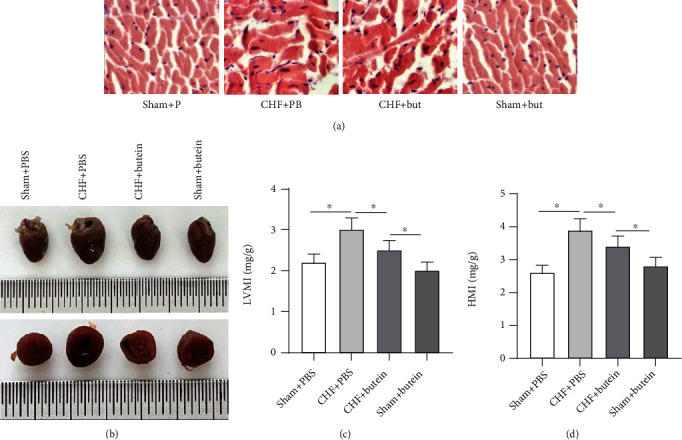
Butein relieves myocardial dysfunction. (a) H&E staining was used to observe the myocardial cell morphology. (b) The corresponding transverse cross-sections at the mid-ventricle level was performed in four groups. (c, d) Representative gross morphology of the whole hearts was expressed by LVMI and HMI after different interventions. ^∗∗^*p* < 0.01.

To determine the histological changes induced by different interventions, we stained the pathological sections with H&E staining to observe the myocardial cell morphology. The sham groups showed normal structure, with uniform distribution of pink cardiomyocytes and interconnection of short columnar cells. The sections of CHF model group exhibited disorders of cardiomyocyte arrangement, compared with the sections in sham operation group. Treatment with butein partially restored the changes and showed a better organization (Figure 4(a)). Rats in all groups were euthanized after left ventricular intubation. Whole hearts were resected followed by measurement of the size and weight of the left ventricle and whole heart. Compared with the sham+PBS group, cardiac volume in CHF+PBS group was grossly enlarged (Figure 4(b)). Compared with CHF+PBS group, the cardiac volume of rats from CHF+butein group was grossly narrow. Consistently, LVMI and HMI were both increased in CHF+PBS group, and the upregulation was then partially reversed in CHF+butein group (Figures 4(c) and 4(d)). Therefore, these results indicated that butein can ameliorate cardiac dysfunction.

### 3.5. Butein Activates the ERK/NRF2 Pathway

**Figure 5:**
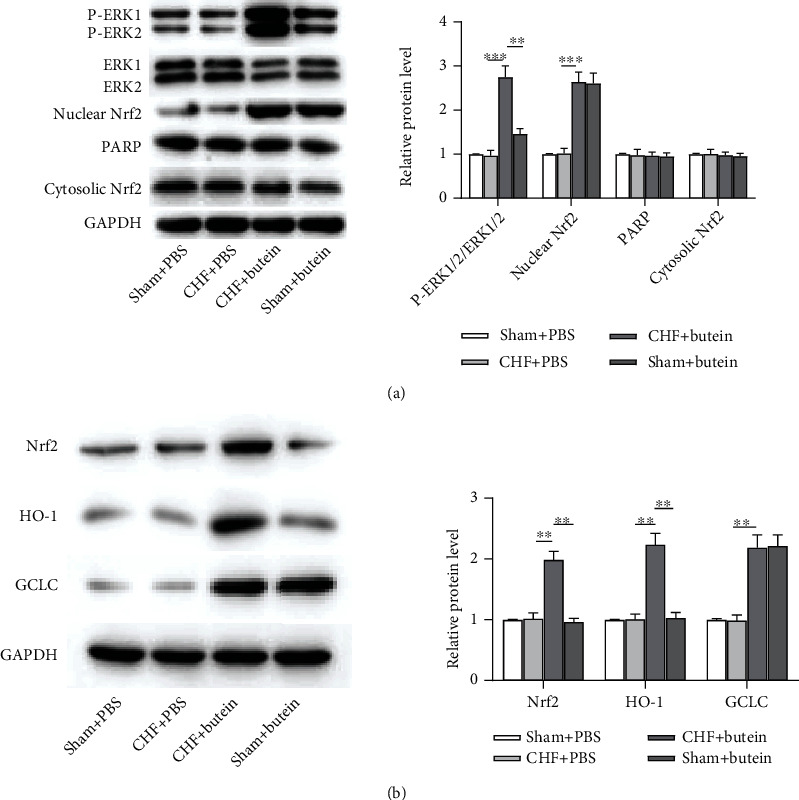
Butein activates the ERK/NRF2 pathway. (a) Level of ERK phosphorylation protein and nuclear accumulation of Nrf2 were examined by western blot analysis. (b) Butein-induced HO-1 and GCLC protein expression was further investigated by Western blot analysis.

According to Western blot analysis, the levels of phosphorylated ERK protein and the nuclear accumulation of Nrf2 were slightly increased in sham+butein, compared to the sham+PBS group. Importantly, butein promoted the level of phosphorylated ERK protein and the nuclear accumulation of Nrf2 in CHF+Butein group, compared with those in the CHF+PBS group (Figure 5(a)). In Figure 5(b), butein significantly induced protein expression levels of Nrf2, HO-1, and GCLC, compared with CHF+PBS group. Interestingly, the expression of phosphorylated ERK, Nrf2, Nrf2, HO-1, and GCLC was significantly upregulated by butein in the sham groups. These data clarified the importance of kinase signaling pathway in the induction of HO-1 and GCLC by butein and implied that ERK1/2 was the candidate kinase for Nrf2 activation, nuclear translocation, and subsequent HO-1 and GCLC induction by butein in both CHF and sham groups.

### 3.6. The Effect of Butein on Oxidative Damage

**Figure 6:**
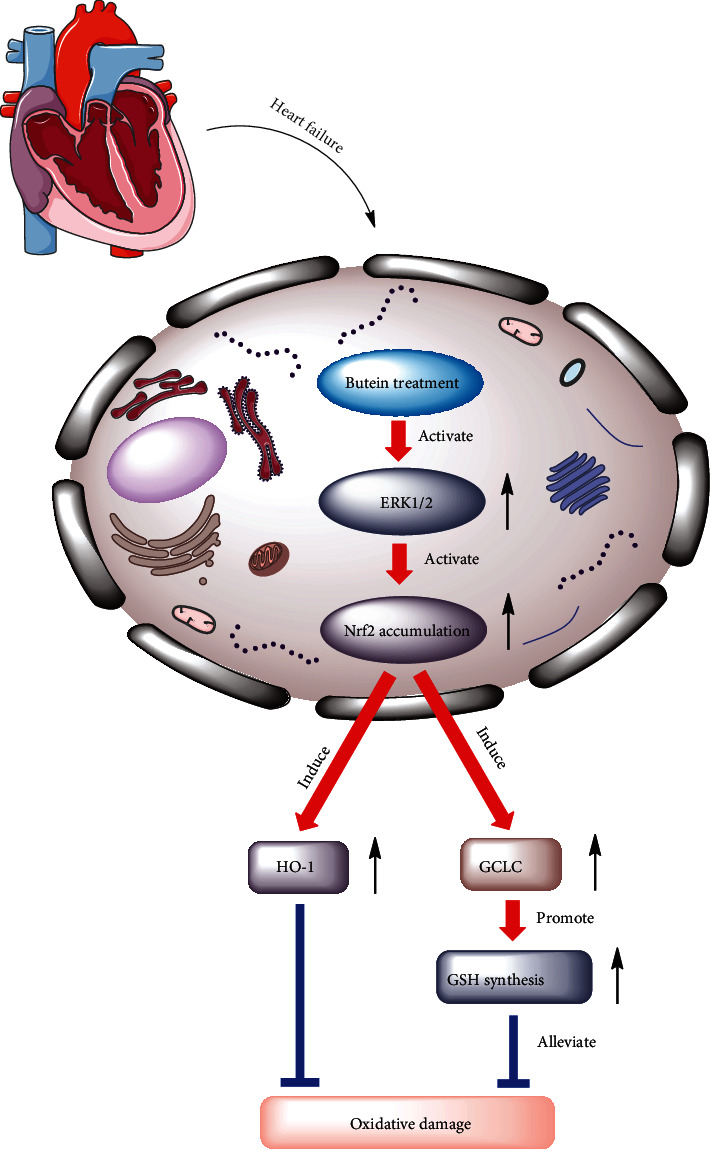
The effect of butein on oxidative damage. The effect of butein on oxidative damage was outlined.

After all the experiments, we outlined the effect of butein on oxidative damage from molecular aspect (Figure 6). Butein significantly upregulated the protein levels of phosphorylated ERK1/2, which contributed to nuclear accumulation of Nrf2. Importantly, Nrf2 is an important antioxidant-regulated transcription factor and exerts its antioxidative role through regulating the protein levels of HO-1 and of GCLC, which resulted in intracellular glutathione (GSH) level increases. Collectively, butein inhibited the oxidative damage in CHF rat via ERK/Nrf2 signaling.

## 4. Discussion

At a cellular level, oxidative stress can induce most of the changes that are thought to contribute to myocardial remodeling myocyte hypertrophy and apoptosis [34]. Furthermore, increasing experimental evidence supports the concept that the myocardial remodeling that leads to heart failure is due the excessive oxidative stress [35, 36]. Thus, the reduction of oxidative molecules and the elevation of antioxidant substance can attenuate myocyte injury and relieve CHF.

Many traditional Chinese medicines have confirmed to play a role in antioxidation in cardiovascular diseases, like Tongxinluo [37], LongShengZhi [38], and Qiliqiangxin [39]. Butein, a kind of chalcone, has also been shown to have antioxidant capacity. For example, butein effectively reduces superoxide anion production in rat liver microsomes through its inherent antioxidant potential [22]. Butein has been proved to enhance the levels of antioxidant response elements (AREs) and p-JNK [40]. However, the role and mechanism of butein in CHF have not been discussed yet. Thus, we first established a CHF rat model for detecting the effect of butein on cardiac function and oxidative stress in CHF rat. Then, we found that butein exerted a protective effect on the cardiac function of CHF rat. Specifically, butein decreased the level of LVESD and LVEDD and increased LVEF and LVFS levels in CHF rat. Importantly, butein obviously upregulated the content of SOD, GSH-Px, and CAT and downregulated MDA level in CHF rat, suggesting an inhibitory effect on oxidative stress. In addition, the levels of myocardial enzymes CK-MB, LDH, and AST were almost two times higher in CHF group than those in sham operation group, while butein partially revered the changes, which suggested that butein relieved cardiac injury. Butein also restored the disorders of pathological sections in CHF model ameliorated cardiac dysfunction. After confirming the effect of butein, we further detected the possible molecular pathways that affected the behavior of butein.

Recently, nuclear factor- (erythroid-derived 2-) like 2 (Nrf2) was confirmed to exert cytoprotective role in regulating oxidative stress [41]. The MAPK family, especially ERK was the classical antiapoptotic pathways. Notably, ERK is phosphorylated activation and subsequently migrates into the nucleus where it regulates the activity of several transcription promoters related to cellular survival under oxidative stress [42]. It has been reported that ERK can cause nuclear translocation of Nrf2 and subsequently bind to the promoter region of antioxidant enzymes such as HO-1, thus becoming a potential upstream regulator of Nrf2 [43]. ERK/NRF2 also plays a significant role in cardiovascular disease. For example, ERK activated Nrf2/HO-1 signaling pathway to reduce ox-LDL-induced inflammation and oxidative stress in vascular smooth muscle cells [44]. The ERK/Nrf2 pathway is involved in oleanolic acid-induced heme oxygenase-1 expression in rat vascular smooth muscle cells [45]. The ERK/NRF2 signal pathway is upregulated by sulfiredoxin-1 to improve the surviving rate of cardiac progenitor cells under antioxidative stress [46]. Interestingly, in our study, butein significantly upregulated the levels of ERK phosphorylation protein and nuclear accumulation of Nrf2 in CHF rat. Upregulation in the protein levels of HO-1 and GCLC also suggested that Nrf2 exerted its regulatory role in antioxidation. Thus, we concluded that butein inhibited the oxidative damage in CHF rat via ERK/Nrf2 signaling. However, previous study has revealed that different doses of butein exert different effect on oxidative stress [23, 47]; thus, the influence of different doses of butein on CHF rat model will be verified in our future study.

Based on the above evidence, butein had effect on animal models of CHF. In detail, butein inhibited oxidative stress, relieved cardiac injury, and alleviated myocardial dysfunction. The ERK/NRF2 pathway provided the possibility for butein to inhibit oxidative stress injury in CHF. These findings demonstrated the regulatory role of butein in Nrf2 pathway was mediated by the phosphorylation of ERK and its antioxidant role in rats with CHF.

## Data Availability

The datasets used or analyzed during the current study are available from the corresponding author on reasonable request.
